# Satisfaction analysis of 5G remote ultrasound robot for diagnostics based on a structural equation model

**DOI:** 10.3389/frobt.2024.1413065

**Published:** 2024-10-09

**Authors:** Zhi-Li Han, Yu-Meng Lei, Jing Yu, Bing-Song Lei, Hua-Rong Ye, Ge Zhang

**Affiliations:** ^1^ Department of Medical Ultrasound, China Resources and Wisco General Hospital, Wuhan University of Science and Technology, Wuhan, China; ^2^ Medical College, Wuhan University of Science and Technology, Wuhan, China

**Keywords:** 5G remote ultrasound robots, structural equation modeling, satisfaction, telemedicine, examination willingness

## Abstract

**Objectives:**

With the increasing application of 5G remote ultrasound robots in healthcare, robust methods are in critical demand to assess participant satisfaction and identify its influencing factors. At present, there is limited empirical research on multi-parametric and multidimensional satisfaction evaluation of participants with 5G remote ultrasound robot examination. Previous studies have demonstrated that structural equation modeling (SEM) effectively integrates various statistical techniques to examine the relationships among multiple variables. Therefore, this study aimed to evaluate the satisfaction of participants with 5G remote ultrasound robot examination and its influencing factors using SEM.

**Methods:**

Between April and June 2022, 213 participants from Wuhan Automobile Manufacturing Company underwent remote ultrasound examinations using the MGIUS-R3 remote ultrasound robot system. After these examinations, the participants evaluated the performance of the 5G remote ultrasound robot based on their personal experiences and emotional responses. They completed a satisfaction survey using a self-developed questionnaire, which included 19 items across five dimensions: examination efficiency, examination perception, communication perception, value perception, and examination willingness. A SEM was established to assess the satisfaction of participants with the 5G remote ultrasound robot examinations and the influencing factors.

**Results:**

A total of 201 valid questionnaires were collected. The overall satisfaction of participants with the 5G remote ultrasound robot examination was 45.43 ± 11.60, with 169 participants (84%) expressing satisfaction. In the path hypothesis relationship test, the dimensions of examination efficiency, examination perception, communication perception, and value perception had positive effects on satisfaction, with standardized path coefficients of 0.168, 0.170, 0.175, and 0.191. Satisfaction had a direct positive effect on examination willingness, with a standardized path coefficient of 0.260. Significant differences were observed across different educational levels in the dimensions of examination perception, communication perception, value perception, and examination willingness. Participants with different body mass indices also showed significant differences in examination perception; all *p*-values were less than 0.05.

**Conclusion:**

In this study, value perception was identified as the most significant factor influencing satisfaction. It could be improved by enhancing participants’ understanding of the accuracy and safety of 5G remote ultrasound robot examinations. This enhances satisfaction and the willingness to undergo examinations. Such improvements not only facilitate the widespread adoption of this technology but also promote the development of telemedicine services.

## 1 Introduction

Telemedicine uses contemporary communication, electronic, and computer technology to enable the remote gathering, storage, processing, transmission, and retrieval of diverse medical data (Constantinescu et al., 2018). Numerous research findings have indicated that telemedicine has the potential to mitigate imbalances between supply and demand, improve the availability of medical imaging services by bridging geographical barriers, and thereby facilitate the effective and rational allocation of healthcare resources (Zhang J. et al., 2022a). Remote ultrasound is a vital component of telemedicine (Jiang et al., 2023). Telemedicine has advanced in recent decades, and remote ultrasound technology has simultaneously progressed (Wootton, 2001). Studies have demonstrated that remote ultrasound helps in avoiding unnecessary long-distance transportation of patients, saving time and costs, reducing queues and gatherings of individuals, decreasing the cross-transmission of epidemics (Adams et al., 2022a), and enhancing the quality of medical services among many other advantages (Zhang G. et al., 2023a). With the rise of ultrasound machines and the availability of data transmission technologies, the necessary infrastructure is in place to enable the development of remote ultrasound (Britton et al., 2019).

Remote ultrasound robot, an advanced application within the realm of remote ultrasound, integrates robotic technology with remote communication mechanisms (Heer et al., 2001). This system typically comprises a robotic arm capable of remotely controlling an ultrasound probe with precision, alongside a transmission system for the real-time relay of images and data to distant clinicians for analysis and diagnosis (Teng et al., 2022). Over the past 20 years, advancements in communication, master–slave, and human–robot interaction systems have led to the development of remote ultrasonic robot technology for diagnostic applications across an extensive geographical area (Lee et al., 2021). Remote ultrasound robot technology enables sonologists to remotely control ultrasound probes for real-time examinations, expanding ultrasound diagnostics to both local and distant sites and utilizing off-site expert sonography (Adams et al., 2021). Off-site medical information has become genuinely attainable (Steller et al., 2014). Numerous studies have demonstrated that a 5G remote ultrasound robot can decrease the need for patients to travel long distances, saving them time and money (Evans et al., 2020). Previous research has demonstrated the effectiveness of 5G remote ultrasound robots during the coronavirus disease 2019 (COVID-19) pandemic (Wang et al., 2021), in intensive care units (Chai et al., 2022), checkups (Ren et al., 2023) and in the assessment of abdominal (Mahoney et al., 2019), thoracic (Ye et al., 2021), and vascular regions (Jenkins et al., 2022). The use of remote ultrasound robot not only expands the scope of remote ultrasound but also enhances operational flexibility and precision, making remote ultrasonic examinations significantly efficient and effective (Guo et al., 2019).

With the development and application of 5G remote ultrasound robot technology, understanding and evaluating user satisfaction with these advanced medical devices has become particularly important. This assessment not only reveals the current state of user experience but also indicates directions for improvement, thereby driving the optimization of technology and enhancement of service quality. Particularly, in the medical field, as the popularity and demand for remote medical services continue to grow, ensuring that these services meet user expectations and needs has become crucial. Therefore, through an in-depth study of participant satisfaction and its determinants, we can not only evaluate the current status of 5G remote ultrasound robot technology but also provide guidance for future technological development and services (Lu et al., 2023). This ensures that a 5G remote ultrasound robot can better serve society and the medical field, particularly playing a substantial role in primary healthcare settings (Alsabeeha et al., 2023). Previous research has explored the awareness, use, and satisfaction of participants with these robots, along with related individual factors (Vatsvag et al., 2020). Previous studies have demonstrated that the satisfaction rating of patients with telemedicine during the COVID-19 pandemic ranged from 70% to 97.6% (Gwilt et al., 2022). Research carried out by Ren and colleagues revealed that satisfaction was universally reported among participants with the 5G-enabled remote ultrasound robot examinations, with 96% of the individuals indicating an absence of concern related to the robotic arm throughout the diagnostic procedure (Ren et al., 2023). Previous research has indicated that all patients either strongly agreed or somewhat agreed with the statement: should conventional ultrasound examinations not be available in their community, they would be willing to undergo telerobotic scanning in the future (Adams et al., 2017). Previous research has indicated that 95% of patients would be willing to undergo another remote ultrasound robot examination in the future (Adams et al., 2022b). Previous research examining satisfaction with 5G remote ultrasound robots has mainly relied on self-developed questionnaire (Kaminski et al., 2020). However, this method is vulnerable to subjective biases in interpretation, potentially affecting the reliability of the findings (St-Pierre et al., 2015). Furthermore, the satisfaction levels with 5G remote ultrasound robot vary and the influencing factors remain unclear, with a lack of comprehensive empirical research exploring the satisfaction levels and determinants among participants who have experienced examinations with 5G remote ultrasound robot. Studies suggest that satisfaction is likely associated with factors such as examination efficiency. (Li and Wang, 2021). These elements are vital for a highly accurate assessment of user satisfaction with 5G remote ultrasound technology (Anderson and Fornell, 2010).

Factor analysis is a common approach to examine the relationships between influencing factors (Zhang J. et al., 2023b). This method is frequently used in satisfaction research but is prone to errors in measuring subjective variables, which cannot be accurately captured by a single indicator (Jurek et al., 2023). Therefore, using conventional multivariate statistical methods might reduce the accuracy of the model when studying the relationship between such subjective latent variables (Bollen et al., 2022). Structural equation modeling (SEM) is particularly advantageous in scenarios where discrepancy exists between latent and observed variables, offering considerable benefits over conventional methods for studying latent variable relationships (Jha and Singh, 2020). SEM allows for a rigorous, empirical evaluation of research hypotheses using statistical analysis, often employed to validate and refine satisfaction models (Tong et al., 2022). This method enables a comprehensive analysis of abstract concepts, integrating both factor and regression analysis (Oduro et al., 2018). Therefore, SEM can integrate a variety of statistical techniques, including correlational analysis, logistic regression, and confirmatory factor analysis, thereby offering a comprehensive methodological framework.

This study conducted physical examinations and surveys among individuals who have used 5G remote ultrasound robots and established an SEM to analyze their satisfaction and its influencing factors. Participant satisfaction serves as a primary reference for assessing the quality of healthcare services and understanding a participant needs. By comprehending participant satisfaction, enhancing the services provided to them becomes feasible.

## 2 Methods

### 2.1 Subjects

Between April and June 2022, a cohort of 213 participants from Wuhan Automobile Manufacturing Company, located approximately 20 km from China Resources and Wisco General Hospital, participated in a remote ultrasound examination. The inclusion criteria for the study were voluntary acceptance of the remote ultrasound robot examination, adequate cognitive ability to comprehend and complete the survey, and consent to participate in the study. The exclusion criteria encompassed patients who declined participation in the remote ultrasound examinations, those experiencing poor network connectivity during the examination, and instances of frequent interruptions. The research protocol was endorsed by the ethics committees of the participating hospitals, and each participant provided written informed consent prior to involvement.

This examination was performed using a robot system operated by a sonographer having 20 years of ultrasound experience at China Resources and Wisco General Hospital. For the execution of remote ultrasound robot examination, the sonographer received prior training in operating the remote ultrasound robot. Participants were asked to evaluate the performance of the 5G remote ultrasound robot based on their personal experiences and emotional responses. A physical assistant on site was designated to support the 5G remote ultrasound robot during the examination process, and concurrently, research personnel were deployed to administer questionnaires to the participants. Prior to the remote medical examination, the physical assistant on site and research personnel underwent training in operating the robot and administering questionnaires.

### 2.2 Instruments

The remote ultrasound robot system (MGIUS-R3; Mindray Biomedical Electronics Co., Ltd., Shenzhen, China), integral to this study, comprises three primary components: the patient-side system, the physician-side system, and a high-definition video communication system. The system is equipped with two probes: C5-1 and L15-4. The patient-side equipment was stationed at Wuhan Bus Manufacturing Co., Ltd., facilitating remote examinations, and the physician-side system was located at China Resources and Wisco General Hospital.

**FIGURE 1:**
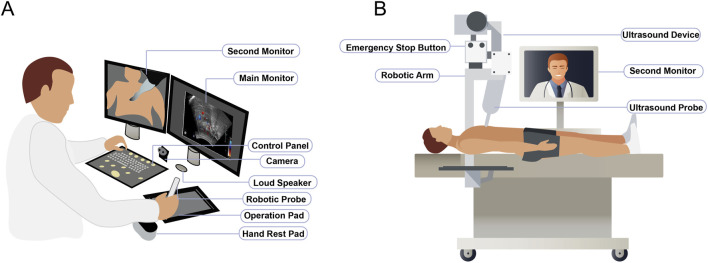
Structural compositions of the remote ultrasound robot system. **(A)** Physician-side system. **(B)** Patient-side system.

The physician-side system integrates mechanical elements, a management system for operations, an audio–visual communication framework, and control software. The mechanical aspect includes an operative plat form responsive to physician directives and capable of relaying feedback from the system at the patient’s end. The operational management system encompasses a robotic probe, operation pad, ultrasound control panel, hand rest pad, and main monitor. The robotic probe in the remote ultrasound robot system is designed to capture the operational positioning and orientation as perceived by the sonographer. The operation pad consolidates data on the position and forces applied by the robotic mechanism at the patient side. Utilizing both the robotic probe and operation pad guarantees the coordination of actions between the physician and the robotic mechanism via established coordinate system transformations. The ultrasound control panel facilitates the adjustment of various ultrasound settings, such as the frequency, amplification, focal adjustment, scanning depth, and dynamic range, enabling real-time image acquisition at the patient side. The hand rest pad provides adequate support for the sonographer’s wrist. The main monitor features a high-definition 1080p medical-grade screen, presenting ultrasound images and relevant data in real time. The audio–visual setup includes a camera, second monitor, and loudspeaker (Figure 1A).

The patient-side system includes mechanical parts, the operation execution system, an ultrasound device, the same audio–visual system, and patient-side control software. The robotic arm in the patient-side system, fitted with an ultrasound probe, receives the physician’s instructions and provides feedback to the physician-side system. This arm is integrated with a highly accurate, adaptable six-dimensional force sensor to obtain real-time feedback when the ultrasound probe interacts with the patient’s soft tissue. The ultrasound system consists of the probe and a device for acquiring, processing, and transmitting ultrasound images. The audiovisual system, identical to that at the physician-side system, facilitates simultaneous communication among sonographers, participants, and assistants. The patient-end control software is responsible for collecting ultrasound images. For safety, the robotic arm’s precise force control sensor and protection algorithm keep speed and pressure within preset limits. During abdominal exams, the vertical and horizontal force ranges are set at 3–40 N and 0–20 N, respectively. If excessive force is detected, the emergency stop button activates, stopping the arm immediately to ensure patient safety (Figure 1B).

### 2.3 Remote ultrasound medical examination

Before the procedure commenced, the on-site physical assistant documented the subject’s details and secured the abdominal ultrasound transducer onto the robotic arm’s probe holder. The subject was positioned supine on the examination table, exposing the abdominal region. The sonographer then adjusted the system to abdominal scan mode and activated the robotic mechanism. The assistant applied a sufficient quantity of ultrasonic coupling gel to the abdominal upper section. Following this, the sonographer controlled the virtual ultrasound probe to direct the patient-side robotic arm to carry out the scan. Ultrasound assessments for male subjects included the thyroid, liver, gallbladder, pancreas, spleen, kidneys, bladder, and prostate, whereas female subjects underwent scans of the thyroid, mammary glands, liver, gallbladder, pancreas, spleen, kidneys, bladder, uterus, and ovaries. Throughout the scanning process, the sonographer could switch the video feed to either observe the patient and their environment or the probe’s orientation and position. Leveraging sophisticated audio–visual communication technologies, the sonographer instructed subjects on positional adjustments and supported the assistant in the application of ultrasonic coupling gel. Following the completion of the ultrasound scans, the sonographer promptly compiled a report detailing the findings, which were subsequently uploaded to a secure online platform for participant access.

### 2.4 Questionnaire survey

Employing the American customer satisfaction index (ACSI) framework, this study developed a hypothetical model to evaluate participant satisfaction with a 5G remote ultrasound robot. A comprehensive ACSI-based model, encompassing five dimensions, was utilized to investigate the determinants of participant satisfaction with the remote ultrasound robot technology. The questionnaire, designed through an extensive literature review, consultations with experts (including 5G ultrasound robot researchers, operators, and clinical ultrasound physicians), and specialized research groups, was tailored to the ACSI model and specific attributes of 5G remote ultrasound robot. The survey instrument evaluated five primary dimensions: examination efficiency, examination perception, communication perception, value perception, and examination willingness. An additional item gauged overall satisfaction, resulting in a total of 19 questions. Each specific question within each dimension is clearly presented in Table 1. Responses were measured on a five-point Likert scale, ranging from 1 (strongly agree) to 5 (strongly disagree).

**TABLE 1:** Descriptive analysis of the dimensions of the questionnaire and the scores of each item.

Dimensionality	Variable ID	Item	Mean ± SD
Examination efficiency			7.16 ± 3.81
	Q1	Do you think the duration of a 5G remote ultrasound robot examination is long?	2.34 ± 1.35
	Q2	Do you think the process of a 5G remote ultrasound robot examination is complicated?	2.37 ± 1.41
	Q3	Do you think the preparation for a 5G remote ultrasound robot examination is adequate?	2.44 ± 1.40
Examination perception			10.11 ± 2.99
	Q4	Do you feel significant pressure from the probe during a 5G remote ultrasound robot examination?	2.55 ± 0.85
	Q5	Do you experience significant pain from the probe during a 5G remote ultrasound robot examination?	2.56 ± 0.87
	Q6	Do you feel significant injury from the probe during a 5G remote ultrasound robot examination?	2.52 ± 0.87
	Q7	Do you feel significant anxiety during a 5G remote ultrasound robot examination?	2.49 ± 0.88
Communication perception			7.76 ± 3.81
	Q8	Is your communication with the consulting specialist at the physician-side during a 5G remote ultrasound robot examination smooth?	2.53 ± 1.31
	Q9	Is your communication with the on-site support staff during a 5G remote ultrasound robot examination smooth?	2.64 ± 1.38
	Q10	Is it common for interruptions or issues affecting the examination to occur during a 5G remote ultrasound robot examination?	2.58 ± 1.37
Value perception			9.77 ± 4.79
	Q11	Do you think the results of a 5G remote ultrasound robot examination are accurate?	2.51 ± 1.41
	Q12	Do you trust the technology of a 5G remote ultrasound robot examination?	2.46 ± 1.38
	Q13	Do you think 5G remote ultrasound robot examination can develop and be widely promoted effectively?	2.40 ± 1.38
	Q14	Do you think 5G remote ultrasound robot examination is safe?	2.40 ± 1.40
Examination willingness			8.58 ± 3.47
	Q15	Do you think 5G remote ultrasound robot examination will replace conventional ultrasound examinations?	2.17 ± 1.01
	Q16	If you need another ultrasound examination in the future and your community does not have an ultrasound examination, are you willing to undergo a robot remote scan?	2.14 ± 0.96
	Q17	Would you recommend 5G remote ultrasound robot examination to your friends?	2.20 ± 0.94
	Q18	Would you recommend 5G remote ultrasound robot examination to people around you?	2.06 ± 0.94
Satisfaction	Q19	What is your overall satisfaction with this 5G remote ultrasound robot examination?	2.14 ± 0.79
Total score			45.43 ± 11.60

Note: Mean ± SD, is the mean ± standard deviation of the dimension or entry scores, and the formal questionnaire has 19 items with a total score of 95.

Examination efficiency assessed the participant’s evaluation of the procedural efficiency during the robotic medical examination. Examination perception involved a general assessment of the 5G remote ultrasonic robot’s inspection service. Communication perception during the check pertained to the evaluation of interactions with doctors and assistants. The value perception of ultrasound robot encompassed the participant’s subjective assessment of the robot’s status, effectiveness, and development, along with their value perception realization relative to time investment. Examination willingness reflected the participants’ overall attitude or behavior toward the device, based on their comprehensive screening experience. In addition to these variables, the study also gathered basic demographic data of the participants, including age, gender, education level, and body mass index (BMI) classification. The details of the questionnaire are presented in Table 1.

Upon conclusion of the remote ultrasound examination, the research personnel provided the participants with a questionnaire and asked them to sign an informed consent form. The participants were instructed to complete the questionnaire based on their actual experiences. During the questionnaire survey, the research personnel were responsible for ensuring the data quality of the questionnaire, including verifying its completeness and preventing duplicate entries. However, the research personnel did not interfere with the specific content of the questionnaire.

### 2.5 Structural equation modeling

In the present investigation, factor analysis was employed to categorize 19 questions designed to assess participant satisfaction into specific dimensions. Subsequently, the reliability and validity of the questionnaires measuring participant satisfaction were examined. The instrument’s reliability was determined through the calculation of Cronbach’s alpha coefficient, which is a statistical measure used to assess the internal consistency of the scale, indicating how closely related a set of items are as a group. To compute Cronbach’s alpha, we calculated the average inter-item correlation among the questions within each dimension, thereby evaluating how well the items measure the same underlying construct. A higher Cronbach’s alpha value (closer to 1) reflects greater internal consistency, suggesting that the items are sufficiently correlated to represent the construct reliably. Cronbach’s alpha provides preliminary evidence of internal consistency, which is a crucial prerequisite for conducting validity analysis in SEM. In this study, after establishing the reliability of the scale, SEM was employed to comprehensively assess the validity of the satisfaction measurement tool and analyze the structural relationships among the identified latent variables. This approach allowed for a more thorough validation of the measurement model and the theoretical assumptions underpinning the study.

Among the variables, examination efficiency, examination perception, communication perception, and value perception were the exogenous latent variables, whereas satisfaction and examination willingness were the endogenous latent variables. The following hypotheses were formulated on the basis of the relationships among the five dimensions and their associations with the ACSI framework:

H1 Examination efficiency has a positive impact on satisfaction.

H2 Examination perception has a positive impact on satisfaction.

H3 Communication perception has a positive impact on satisfaction.

H4 Value perception has a positive impact on satisfaction.

H5 Satisfaction has a positive impact on examination willingness.

Within the analytical structure of the study, SEM was utilized to assess the impact of participant attributes on their satisfaction. SEM consists of two sub-models: measurement and structural. The measurement model is a representation of the theory that outlines how measured variables are combined to reflect the underlying theory. It can be formulated as

$$x=\Lambda_{x}\xi +\delta ,$$

$$y=\Lambda_{y}\eta +\varepsilon ,$$

where x is the measured variable for the exogenous latent construction of ξ, y is the measured variable for the endogenous latent construction of η, and Λ_x_ and Λ_y_ are the factor loadings of x and y, respectively. δ and ε are the measurement errors in x and y, respectively. A structural model is a theoretical framework that illustrates the relationships between different constructs. It can be formulated as

$$\eta =B_{\eta }+\Gamma \xi +\zeta .$$

Here, B and Γ denote the coefficient matrices for the endogenous and exogenous latent constructs, respectively. The term ζ refers to the residuals, which cannot be expressed or predicted.

The standards for SEM model fit indices are usually as follows: the values of goodness-of-fit index (GFI), adjusted goodness-of-fit index (AGFI), normed fit index (NFI), comparative fit index (CFI), incremental fit index (IFI), and Tucker–Lewis index (TLI) should be close to or greater than 0.90, indicating good model fit. The value of root mean square error of approximation (RMSEA) should be less than 0.08, ideally less than 0.05, indicating a low error of approximation. The Chi-square (χ^2^) to degrees of freedom ratio should be less than 3, indicating a good model fit. In SEM, the standard error (S.E.) quantifies the precision of parameter estimates; a smaller S.E. signifies greater precision in the estimation. The critical ratio (C.R.) is derived from the ratio of the parameter estimate to its S.E. value and is utilized to evaluate the statistical significance of the parameter. A C.R. value with an absolute magnitude exceeding 1.96 typically indicates that the parameter is statistically significant, thus affirming its relevance in the model.

### 2.6 Statistical analysis

To elucidate the core attributes of the individuals, the research team compiled statistical data including frequency (N) and percentage (%). To examine disparities in satisfaction levels across individuals with different characteristics, a one-way analysis of variance was employed. Additionally, mean (M) and standard deviation (SD) values were calculated to assess varying degrees of satisfaction related to the 5G remote ultrasound robot. To ensure scientific rigor and methodological appropriateness, this study utilized datasets containing both categorical and continuous variables. The continuous data approximately followed a normal distribution, rendering them suitable for parametric tests. The analysis was based on a survey designed to assess participant satisfaction across multiple dimensions. Such research is ideally suited to methods capable of addressing the complex relationships between observed and latent variables. To evaluate the reliability of the measurement items, Cronbach’s alpha—a widely recognized indicator of internal consistency—was employed. Additionally, exploratory factor analysis (EFA) was performed to identify potential factors, followed by confirmatory factor analysis (CFA) to validate the factor structure. This approach ensured the robustness and validity of the measurement tool, thereby providing a solid foundation for subsequent analyses (Bollen et al., 2022).

SEM is deemed an optimal method for examining latent variable relationships owing to its capability to simultaneously handle multiple datasets and model causal relationships (Sahin et al., 2006). Compared to traditional multivariate statistical techniques, such as regression and factor analyses, SEM affords a more precise approach to addressing the interactions between latent and observed variables. To elucidate the pathways and interrelationships among the dimensions, SEM was employed. For parameter estimation within the SEM framework, maximum likelihood estimation was utilized. Statistical analyses were conducted using Statistical Package for the Social Sciences (SPSS) version 26.0 and Analysis of Moment Structures (AMOS) version 7, both from SPSS Inc., Chicago, IL, USA. Statistical significance was determined with a threshold of *p* < 0.05. Given the presence of latent variables, such as satisfaction and examination willingness, SEM was adopted owing to its applicability in modeling complex relationships among latent and observed variables. To evaluate the fit of the SEM model, several fit indices were used, including the GFI, NFI, TLI, and RMSEA, ensuring that the model adequately represents the data. Additionally, Principal Component Analysis (PCA) and Varimax rotation were employed to extract and rotate factors, ensuring that the factors were uncorrelated and interpretable. Adhering to these rigorous criteria and methods, we ensured the robustness and appropriateness of our statistical analysis.

## 3 Results

### 3.1 Reliability and validity of measurement items

We observed high reliability in our measurement items, as evidenced by Cronbach’s alpha values. The overall reliability of the study, indicated by a Cronbach’s alpha of 0.84, significantly exceeded the standard reliability threshold of 0.6. The Cronbach’s alpha values for examination efficiency and examination perception were 0.91 and 0.88, respectively. The communication perception demonstrated an even higher reliability with a Cronbach’s alpha of 0.94. The perceptions of value and examination willingness yielded Cronbach’s alpha values of 0.88 and 0.92, respectively. All latent variables in this survey showed Cronbach’s alpha values ranging from 0.88 to 0.94, affirming the reliability of the data. Additionally, the Kaiser–Meyer–Olkin (KMO) value for this study was 0.82. Bartlett’s test of sphericity revealed a χ^2^ value of 2,608.38 with a significance level of *p* < 0.01 (*p* = 0.00), demonstrating the appropriateness of the data for factor analysis. Bartlett’s test of sphericity yielded a significance level of *p* < 0.01. By using principal component extraction and varimax rotation, this study identified five distinct common factors, representing key dimensions essential for a comprehensive understanding of the overall evaluation of the remote ultrasound robotic system. The cumulative variance of these factors amounted to 81.1%. This implies that the five extracted common factors can explain 81.1% of the total variance in the data. This cumulative variance value is well above the commonly accepted standard of 60% in social science research (Vaezi et al., 2019). These findings demonstrate that the variables formulated for this investigation have substantial construct validity, thereby reinforcing the robustness and credibility of our research approach.

**FIGURE 2:**
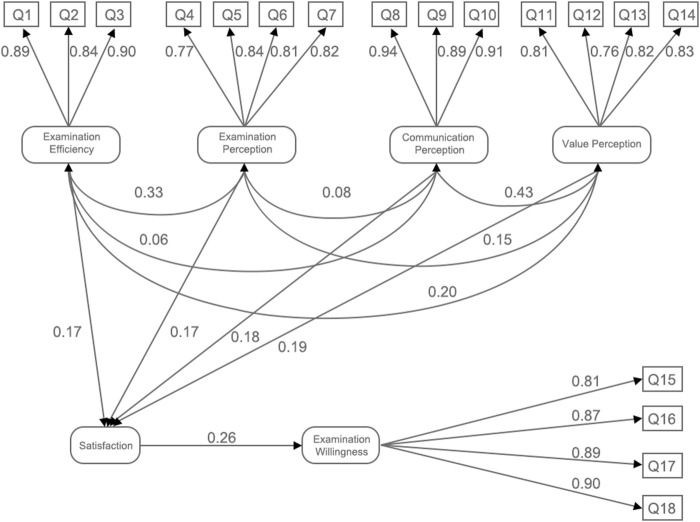
Path relationships among the five dimensions of satisfaction with 5G remote ultrasound robot.

The standardized factor loadings for each question of examination efficiency are 0.89, 0.84, and 0.90. And the standardized factor loadings of examination perception, communication perception, value perception and examination willingness were 0.77–0.84, 0.89–0.94, 0.76–0.83, and 0.81–0.90, respectively (see Figure 2). Standardized factor loadings with values exceeding 0.70 are considered exceptionally robust, indicating a strong correlation between the observed variables and latent constructs.

### 3.2 Basic characteristics of participants

A total of 213 people were surveyed, 202 questionnaires were collected, and one unqualified questionnaire was excluded, resulting in 201 valid questionnaires, with an efficiency rate of 99%. The participants comprised 108 males (54%) and 93 females (46%). From the distribution of education structure, the education level was mostly bachelor’s degree, and the dominant age group was 34–43 years with 104 people, accounting for 51%. The survey respondents and satisfaction scores are presented in Table 2, and the test of variance indicates that BMI classification (F = 1.45, *p* = 0.04) is a significant predictor of satisfaction (Table 2). Variance analysis of the five dimensions across different educational levels and BMI groups revealed significant differences. Specifically, participants with higher educational attainment reported significantly greater satisfaction in examination perception (F = 3.77, *p* < 0.05), communication perception (F = 3.41, *p* < 0.05), and examination willingness (F = 3.13, *p* < 0.05). Conversely, participants with education levels at the secondary school level or below exhibited higher satisfaction regarding the value perception. In terms of BMI, participants with a 24.0–27.9 BMI had the highest satisfaction in value perception (F = 3.13, *p* < 0.05) (Table 3).

**TABLE 2:** Descriptive statistics of participants’ characteristics.

Variable	Characteristics	Number (N)	Percentage (%)	Mean ± SD	F	*p*
Gender	Male	108	53.7	45.82 ± 11.01	0.96	0.55
	Female	93	46.3	44.97 ± 12.28		
Age	18∼33	50	24.9	43.54 ± 11.62	1.27	0.14
	34∼43	104	51.7	46.29 ± 11.65		
	44∼53	44	21.9	45.82 ± 11.78		
	54∼65	3	1.5	41.33 ± 3.79		
Education level	Junior high school and below	9	4.5	46.33 ± 15.75	0.88	0.69
	High school or college	48	23.9	46.46 ± 10.65		
	Undergraduate	96	47.8	46.00 ± 12.34		
	Graduate student and above	48	23.9	43.08 ± 10.07		
BMI classification	<18.5	12	6.0	48.33 ± 15.44	1.45	0.04
	18.5∼23.9	109	54.2	46.10 ± 11.65		
	24.0∼27.9	69	34.3	43.72 ± 10.76		
	>28.0	11	5.5	46.27 ± 11.75		

Note: Mean ± SD, represents the mean and standard deviation of the total score (95).

**TABLE 3:** Analysis of the variability of each dimension of the questionnaire for different educational levels and BMI.

Dimensionality	Education level	N	Mean ± SD	F	*p*	BMI	N	Mean ± SD	F	*p*
Examination efficiency	Junior high school and below	9	7.22 ± 3.35	0.57	0.64	<18.5	12	7.42 ± 4.21	0.48	0.70
	High school or college	48	6.71 ± 3.46			18.5–23.9	109	7.42 ± 3.91		
	Undergraduate	96	7.51 ± 3.93			24.0–27.9	69	6.75 ± 3.59		
	Graduate student and above	48	6.90 ± 4.02			>28.0	11	6.82 ± 3.95		
	Total	201	7.16 ± 3.81			Total	201	7.16 ± 3.81		
Examination perception	Junior high school and below	9	9.67 ± 1.50	3.77	0.01	<18.5	12	12.25 ± 3.57	3.13	0.03
	High school or college	48	11.27 ± 3.04			18.5–23.9	109	10.14 ± 2.89		
	Undergraduate	96	9.97 ± 3.23			24.0–27.9	69	9.58 ± 2.83		
	Graduate student and above	48	9.33 ± 2.31			>28.0	11	10.91 ± 3.51		
	Total	201	10.11 ± 2.99			Total	201	10.11 ± 2.99		
Communication perception	Junior high school and below	9	8.89 ± 3.79	3.41	0.02	<18.5	12	6.58 ± 4.23	1.79	0.15
	High school or college	48	8.77 ± 3.88			18.5–23.9	109	7.90 ± 3.82		
	Undergraduate	96	7.58 ± 3.92			24.0–27.9	69	7.16 ± 3.68		
	Graduate student and above	48	6.46 ± 3.15			>28.0	11	9.55 ± 3.45		
	Total	201	7.66 ± 3.80			Total	201	7.66 ± 3.80		
Value perception	Junior high school and below	9	8.11 ± 6.25	3.24	0.02	<18.5	12	11.17 ± 5.62	1.25	0.29
	High school or college	48	8.17 ± 3.24			18.5–23.9	109	9.92 ± 4.73		
	Undergraduate	96	10.24 ± 5.05			24.0–27.9	69	9.67 ± 4.95		
	Graduate student and above	48	10.75 ± 4.91			>28.0	11	7.45 ± 2.58		
	Total	201	9.77 ± 4.79			Total	201	9.77 ± 4.79		
Examination willingness	Junior high school and below	9	10.56 ± 5.66	3.13	0.03	<18.5	12	8.75 ± 3.55	0.24	0.87
	High school or college	48	9.27 ± 3.55			18.5–23.9	109	8.59 ± 3.69		
	Undergraduate	96	8.57 ± 3.51			24.0–27.9	69	8.42 ± 3.22		
	Graduate student and above	48	7.54 ± 2.47			>28.0	11	9.36 ± 2.94		
	Total	201	8.58 ± 3.47			Total	201	8.58 ± 3.47		
Satisfaction	Junior high school and below	9	1.89 ± 0.60	0.78	0.51	<18.5	12	2.17 ± 0.94	0.01	1.00
	High school or college	48	2.27 ± 0.76			18.5–23.9	109	2.14 ± 0.75		
	Undergraduate	96	2.13 ± 0.82			24.0∼27.9	69	2.14 ± 0.81		
	Graduate student and above	48	2.10 ± 0.78			>28.0	11	2.18 ± 0.98		
	Total	201	2.14 ± 0.79			Total	201	2.14 ± 0.79		

### 3.3 Scores of five dimensions by characteristics

A statistical analysis of the total scores for all 19 questions from the 201 questionnaires yielded an average score of 45.43 with an SD of 11.60. It is worth noting that the questionnaire design contains 19 questions, with a total score of 95 points, and the scoring mechanism is reversed, that is, the lower the score, the higher the satisfaction of the participants.

This study revealed that the overall satisfaction among the participants was 84%. In this study, satisfaction was clearly defined as achieving at least 50% of the highest possible score on the questionnaire (95 points), i.e., achieving at least 47.5 points. However, the actual average score was lower than this threshold, indicating that participants were generally more satisfied with the 5G remote ultrasound robotic physical examination, as their scores were well below the theoretical “unsatisfactory” boundary.

Participants’ scores for examination efficiency, examination perception, communication perception, value perception, and examination willingness were 7.16 (total 15), 10.11 (total 20), 7.76 (total 15), 9.77 (total 20), and 8.58 (total 20), respectively. The proportion of scores across various dimensions shows that the average score for examination willingness was the lowest, indicating the highest level of satisfaction. This suggests that participants were generally willing to undergo remote robotic ultrasound examinations and were inclined to recommend them to others. Conversely, the average score for examination perception was the highest, indicating dissatisfaction with aspects such as probe pressure during the examination and a tendency towards anxiety related to the robotic examination. The item “Was your communication with the on-site support staff during the 5G remote ultrasound robotic examination smooth?” received the highest score (2.64 ± 1.38), indicating that communication with on-site support staff during the examination was not entirely smooth. Conversely, the item “Would you recommend 5G remote ultrasound robotic examinations to people around you?” received the lowest score (2.06 ± 0.94), suggesting that participants were generally receptive to the robotic technology and willing to endorse its use. Detailed information is presented in Table 1.

### 3.4 Analysis of satisfaction path in five dimensions

The GFI, AGFI, NFI, CFI, IFI were greater than 0.9. The model fit indices of the SEM were all within specifications (Sahin et al., 2006) (GFI = 0.900, TLI = 0.978, IFI = 0.982, CFI = 0.921, NFI = 0.935, RMSEA = 0.041), and the χ^2^ to freedom degree ratio was 1.329, indicating a good model fit. The model fit indices of the SEM were all within specifications (Hu and Bentler, 1999) (GFI = 0.900, TLI = 0.978, IFI = 0.982, CFI = 0.921, NFI = 0.935, RMSEA = 0.041), and the χ^2^ to freedom degree ratio was 1.329, indicating a good model fit.

The SEM was utilized to evaluate the research hypotheses; the results are detailed in Table 4. The analysis of path hypotheses revealed that examination efficiency, examination perception, perception of examination communication, and perception of value positively influenced satisfaction, with standardized path coefficients of 0.168, 0.170, 0.175, and 0.191, respectively. Furthermore, satisfaction demonstrated a direct positive effect on examination willingness, with a standardized path coefficient of 0.259. The S.E. values ranging from 0.046 to 0.080 indicate a relatively high precision in the estimation of these regression coefficients. Furthermore, all C.R. values exceeded the threshold of 1.96, with corresponding *p*-values being less than 0.05, signifying that the regression coefficients for these paths are statistically significant (Figure 2). This underscores that all specified paths have a significant effect on either satisfaction or examination willingness. Among the dimensions, perception of value had the most substantial impact on satisfaction, as indicated by its standardized path coefficient of 0.191.

**TABLE 4:** Path estimation of the model.

Path	Regression weights	Standardized regression weights	S.E.	C.R.	*p*
Examination efficiency → Satisfaction	0.106	0.168	0.046	2.294	0.02
Examination perception → Satisfaction	0.184	0.170	0.080	2.309	0.02
Communication perception → Satisfaction	0.112	0.175	0.048	2.345	0.01
Value perception → Satisfaction	0.129	0.191	0.054	2.420	0.01
Satisfaction → Examination willingness	0.278	0.259	0.077	3.625	0.00

Note: S.E.: Standard Error. C.R.: critical ratio.

## 4 Discussion

This study examined 213 participants using 5G remote ultrasound robot and investigated their satisfaction and its influencing factors using a self-developed questionnaire. Based on the hypothetical model of ACSI and the results of SEM, this study provided valuable insights into evaluation of participant satisfaction with 5G remote ultrasound robot. The questionnaire was designed in order to balance comprehensiveness with the effective collection of data. Five dimensions were set up to capture a broad spectrum of participant experiences and perceptions, ensuring representation across diverse respondent backgrounds. In order to ensure the depth and specificity of the collected data, the most representative sub-questions were set for each of the five dimensions by integrating relevant literature and expert feedback. The results indicated that the questionnaire had high validity and reliability, the multidimensional model effectively represented satisfaction and its influencing factors, and the hypothesized paths held true. This study revealed that the overall satisfaction among the participants was 84%. Satisfaction was the highest for examination willingness and the lowest for examination perception. The factor exerting the greatest influence on satisfaction was the value perception. Examination efficiency, examination perception, communication perception, and value perception contributed significantly to overall satisfaction with 5G remote ultrasound robots. Additionally, satisfaction was observed to directly influence examination willingness. Variance analysis indicated that individuals with varying educational backgrounds exhibited significant differences in their examination perception, communication perception, value perception, and examination willingness. Similarly, subjects with different BMI exhibited considerable variation in their examination perception.

This study showed that 84% of participants are satisfied with 5G remote ultrasound robots, but this is lower than the level of satisfaction reported in previous studies. Bhuva et al. observed the greatest satisfaction percentage of 97.6% in their study (Bhuva et al., 2020). A previous study indicated that 99% of participants were satisfied or extremely satisfied with the professionalism and service of the ultrasound specialist (Li et al., 2022). A remarkable 98% of subjects reported comfort during abdomen and urogenital examinations; 92.8% of patients reported no severe discomfort and expressed comfort when utilizing the audio–visual communication system to interact with the tele-doctors (Zhang et al., 2022b). The discrepancies between the findings of this study and previous research are primarily because the satisfaction levels reported in earlier studies were derived from simplified questionnaire surveys, which inherently possess subjectivity that could lead to inaccurate assessments. The differences in the results reported by these studies could also be attributed to variations in research methodologies and sample sizes (Zhang et al., 2022c). The highest satisfaction was reported for examination willingness, which could be closely associated with the convenience offered by the 5G remote ultrasound robot. This convenience includes reduced medical visits and alleviated stress of visiting hospitals (McBeth et al., 2011), and it may also relate to the participants’ confidence in the anticipated medical outcomes. This implies that participants believe the 5G remote ultrasound robot can provide accurate and effective diagnostic results (Priester et al., 2013). As an emerging technology, the 5G remote ultrasound robot offers participants a novel medical experience, which itself could be a significant factor contributing to the high level of satisfaction. Satisfaction was the lowest for examination perception, possibly reflecting a discrepancy between the participants’ actual experience with 5G remote ultrasound robot technology and their prior expectations. This discrepancy could stem from overly optimistic expectations regarding the technology’s performance, diagnostic accuracy, or procedural workflow, which the actual experience failed to fully meet. A low satisfaction underscores the significance of user experience in the context of remote medical technology, including aspects such as ease of use, the intuitiveness of the user interface, and immediacy of feedback (Lofgren et al., 2009). Difficulties in understanding or operating the system during use could directly impact the satisfaction levels.

This study demonstrated that examination efficiency positively affects satisfaction, indicating that simple procedures, adequate examination, and higher examination efficiency correlate with higher participant satisfaction. Previous research reported that the examination duration for remote ultrasound robot is longer than that for conventional ultrasound (Adams et al., 2017). The extended examination durations may be attributed to subjects frequently changing positions during examinations of different body parts, the necessity of on-site assistants, and the time spent manually adjusting the original position of the probe after its activation (Adams et al., 2017). However, the use of remote ultrasound robot diagnostic systems for ultrasound examinations, in comparison with conventional ultrasound, has been demonstrated to reduce the overall time cost (Lofgren et al., 2009). For instance, those in good health are spared from having to make the lengthy trip to a local hospital to receive an ultrasound scan. The remote ultrasound robot examination saves the overall examination time cost of the patient, and thus obtains a high degree of satisfaction.

This study indicated that the dimension of examination perception has a positive impact on satisfaction. Previous studies have highlighted concerns regarding the ability of the robotic arm’s force feedback to consistently maintain contact force (Delestrain et al., 2023). This could potentially impact the participants’ perceptual experience while undergoing the examination. Previous studies have indicated that a small number of individuals experience notable neck pain or a sensation of being unable to breathe, particularly in the area surrounding the trachea. The potential cause for this is the insufficient presence of subcutaneous fat to absorb the impact of the ultrasonic transducer. Owing to the natural cylindrical curvature of the neck, the initial sonographer cannot achieve complete mastery of the fit between the US transducer and the neck (Tsumura et al., 2024). Previous studies have demonstrated that 7.2% of patients reported discomfort or suffocating sensations, especially around the trachea, whereas 10.8% of patients reported fear when the robotic arm moved about the neck area (Zhang et al., 2023c). This is also consistent with the conclusions of the present study. Reducing the sensations of pain and discomfort experienced by participants during the examination process can enhance the examination perception, and then increase the satisfaction of examination.

Regarding the communication perception, although the equipment used in this study has impressive download speeds of around 930 Mbps and upload rates of 130 Mbps, with no noticeable delay (≤200 ms) in the inspection process, the network’s stability is a crucial element. Network fluctuations or intermittent interruptions can cause communication problems (Vaezi et al., 2019). Compatibility problems between the devices or software at the doctor and patient sides can cause communication difficulties. Enhancing the fluidity of communication among participants, consulting physicians, and on-site assistants is also a critical aspect that cannot be overlooked. Compared with conventional face-to-face examinations, examinations conducted via robot and remote technologies may limit aspects of nonverbal interpersonal communication, such as body language and eye contact, which typically contribute to enhancing communication and understanding (Hidalgo et al., 2023). In summary, optimizing network transmission during remote ultrasound examination, training of on-site assistants, and improving communication fluency are crucial for improving overall satisfaction and examination experience. These improvements are significant for advancing user experience and driving the development of the technology.

The value perception had the greatest positive impact on satisfaction. In the context of 5G remote ultrasound robots, the value perception encompasses user evaluations of the accuracy, safety, convenience, and cost-effectiveness of medical services. Enhanced user satisfaction is directly correlated with the perception of high-value services offered by these robots, thereby facilitating the technology’s broader adoption and dissemination (Artandi and Stewart, 2018). Therefore, augmenting the perceived value emerges as a critical element in elevating participant satisfaction and propelling the application of 5G remote ultrasound robot technology. Consistent with the findings of this study, previous studies indicated that the majority of patients exhibit a high level of acceptance toward 5G-based remote robot ultrasound systems and are willing to pay additional fees for the examination (Zhang et al., 2022d). Previous studies indicated that patients appreciate the benefits of remote robot ultrasound as it significantly reduces the need for travel and ensures safety, which can be a critical issue in times of public restrictions on travel and physical interactions, such as during the COVID-19 pandemic (Adams et al., 2022a). In conclusion, improving the value perception of 5G remote ultrasound robot by improving robot safety and accuracy is essential for enhancing participant satisfaction and encouraging wider adoption. As patients increasingly recognize the benefits of reduced travel and enhanced safety—especially during restrictive periods such as the COVID-19 pandemic—highlighting the value of these technologies will play a key role in their successful integration and widespread implementation.

In this study, subjects with lean body types (BMI <18.5 kg/m^2^) expressed lower overall satisfaction compared with individuals with normal body types. This is consistent with previous research findings (Tsumura et al., 2024). The variation in satisfaction levels among individuals with a lean physique may be ascribed to the requirement for greater contact force during the examination process, consequently amplifying the relationship between sensory stimulation and psychological perception (Adams et al., 2022b). Furthermore, individuals with leaner physiques may exhibit increased sensitivity to discomfort or pain during the examination process, in contrast to those with normal body types in which thicker adipose tissue may provide a cushioning effect against pressure (Zhang et al., 2022d). Individuals with postgraduate education levels exhibited higher satisfaction in their perception of examination, examination communication, and examination willingness, and a stronger propensity to advocate for the use of this technology, showing a preference for choosing 5G remote ultrasound robots. This indicates that individuals with higher levels of education are more receptive to novel technologies and are more inclined to use them. By contrast, individuals with education levels of middle-school or lower reported higher satisfaction in their perceived value of remote ultrasound robots compared with those with postgraduate education levels. This difference in perception may stem from the limited academic qualifications of the former group, potentially leading to a lack of objective and comprehensive understanding of the 5G remote ultrasound robot (O’Brien et al., 2022). Therefore, such individuals might notice only a narrow gap between their examination experiences and expectations, leading to subjectively biased evaluations and an inflated sense of value. By contrast, individuals with postgraduate education exhibited better perceptions of the examination, more effective communication, higher willingness to advocate for the use of this technology, and greater support for the device and screening process. Additionally, they showed a higher willingness to opt for the 5G remote ultrasound robot, suggesting that people with higher education levels are more receptive to novel technologies and more inclined to use them (Zhang et al., 2022e). These observations align with the conclusions drawn from previous studies in the field (Binesh and Baloglu, 2023).

Despite the valuable insights provided by this study, it has limitations that warrant further research. One limitation is its relatively small sample size. The participants were mostly employees of central enterprises, which could influence the generalizability of the results to a broader population. The extensiveness of samples and sample area distribution require further improvement. Second, the study may not have adequately considered specific variables, such as disease perception and examination environment, which could play a significant role in the evaluation of the device. Another limitation is the reliance on self-developed questionnaire for data collection, which could introduce recall bias into the findings. Although SEM offers a quantitative analytical framework, the construction of the model and the interpretation of results involve a certain degree of subjectivity. Although the ACSI is a widely recognized framework for measuring satisfaction in government services, its application in the field of 5G remote ultrasonic robot diagnostic satisfaction evaluation represents an unexplored area of research.

## 5 Conclusion

In conclusion, this study developed a theoretical model to assess participant satisfaction with a 5G remote ultrasound robot. In this research, 84% of the participant expressed satisfaction with the 5G remote ultrasound robot examination. The empirical analysis identified key factors influencing client satisfaction, including examination efficiency, examination perception, communication perception, and value perception. These factors are crucial in enhancing participant satisfaction with the use of 5G remote ultrasound robots. The value perception exerts the strongest positive influence on participant satisfaction, making it a pivotal aspect in satisfaction evaluations. To enhance participant perception of the value of 5G remote ultrasonic robots, educational activities and promotional materials can be used to provide detailed information about the technology, including its principles, advantages, and potential benefits. Case studies and clinical data demonstrating the robot’s ability in delivering accurate and safe diagnostics should be presented. Allowing potential users to personally experience the technology can foster understanding and trust. Gathering feedback from patients and doctors who have used the 5G remote ultrasonic robot can provide insights its clinical effectiveness and limitations. Transparency in the technology’s operation and capabilities is essential to build trust. These measures can effectively improve participants’ valuation of 5G remote ultrasonic robots, thereby enhancing acceptance and satisfaction.

## Data Availability

The raw data supporting the conclusions of this article will be made available by the authors, without undue reservation.
